# The Case of the Disappearing Spindle Burst

**DOI:** 10.1155/2016/8037321

**Published:** 2016-03-28

**Authors:** Alexandre Tiriac, Mark S. Blumberg

**Affiliations:** ^1^Department of Psychological & Brain Sciences, The University of Iowa, Iowa City, IA 52242, USA; ^2^DeLTA Center, The University of Iowa, Iowa City, IA 52242, USA; ^3^Department of Biology, The University of Iowa, Iowa City, IA 52242, USA

## Abstract

Sleep spindles are brief cortical oscillations at 10–15 Hz that occur predominantly during non-REM (quiet) sleep in adult mammals and are thought to contribute to learning and memory. Spindle bursts are phenomenologically similar to sleep spindles, but they occur predominantly in early infancy and are triggered by peripheral sensory activity (e.g., by retinal waves); accordingly, spindle bursts are thought to organize neural networks in the developing brain and establish functional links with the sensory periphery. Whereas the spontaneous retinal waves that trigger spindle bursts in visual cortex are a transient feature of early development, the myoclonic twitches that drive spindle bursts in sensorimotor cortex persist into adulthood. Moreover, twitches—and their associated spindle bursts—occur exclusively during REM (active) sleep. Curiously, despite the persistence of twitching into adulthood, twitch-related spindle bursts have not been reported in adult sensorimotor cortex. This raises the question of whether such spindle burst activity does not occur in adulthood or, alternatively, occurs but has yet to be discovered. If twitch-related spindle bursts do occur in adults, they could contribute to the calibration, maintenance, and repair of sensorimotor systems.

## 1. Introduction

The cerebral cortex of mammals, including humans, displays oscillatory spindle activity (10–15 Hz) across the lifespan. In adults, sleep spindles, a specific type of spindle activity, occur exclusively during non-REM sleep [1–4] and have been implicated in memory retention and skill learning [5–7]. In humans, sleep spindles first appear 4–9 weeks postterm and become more prominent and frequent over the next several months [2].

There exists another type of spindle activity—called spindle bursts—that is phenomenologically similar to sleep spindles in that they share a similar frequency range, duration, and spindle-shaped waveform [8]. However, spindle bursts differ from sleep spindles in a variety of ways. For example, spindle bursts predominate early in development: they are readily observed in premature human infants [9] and are thought to disappear soon after birth [2]. In rats, spindle bursts have been recorded in sensorimotor and visual cortex soon after birth through at least the end of the second postnatal week [8, 10, 11]. In addition, unlike sleep spindles, spindle bursts are not exclusive to non-REM sleep but rather can occur across the sleep-wake cycle [2]. Finally, spindle bursts are most closely associated with self-generated or evoked activity in the sensory periphery [10]; for example, manually stimulating the limb of a rat or human, during sleep or wakefulness, elicits spindle bursts in sensorimotor cortex [8, 9, 11, 12].

Across all sensory modalities, however, self-generated activity in the sensory periphery is the predominant trigger of spindle bursts during early development. In the visual system, for example, retinal ganglion cells fire spontaneously before eye opening, producing waves of retinal activity that provide downstream afferent input to visual brain areas [10, 13, 14]. In the auditory system, cochlear spiral ganglion cells fire bursts of activity before the onset of hearing that provide downstream afferent input to the auditory system (although spindle bursts have not yet been recorded in auditory cortex, it is likely that they occur) [15–17]. By providing substantial sensory experience during early development, retinal waves and cochlear bursts shape and refine functional links between the sensory periphery and developing brain networks [14, 18, 19].

In the developing sensorimotor system, spontaneous activity takes the form of myoclonic twitching, which is characterized by jerky movements of the forelimbs and hindlimbs, tail, whiskers, and eyes during REM sleep [8, 9, 11, 20, 21]. In neonatal rats, twitches are produced by the red nucleus and other brainstem premotor structures [22]. Sensory feedback from twitching limbs triggers activity throughout the nervous system and has been hypothesized to contribute to the development of sensorimotor circuits [8, 11, 20, 22, 23]. Moreover, just as retinal waves trigger spindle bursts in visual cortex [10], sensory feedback from twitching limbs (i.e., reafference) triggers spindle bursts in somatosensory [8, 24–26] and motor [11, 12] cortex. Twitches, however, differ from retinal waves in two fundamental ways: first, although retinal waves are transient features of development [27], twitches persist into adulthood [28, 29] and second, although retinal waves seem to occur independently of behavioral state, twitches are an exclusive feature of REM sleep [30].

To reiterate, twitches trigger spindle bursts in sensorimotor cortex [8, 11, 12, 24, 25], occur exclusively during REM sleep [30], originate prenatally [9, 31], and persist into adulthood [28, 29]. Curiously, however, spindle bursts have not been reported during REM sleep in adults. One possible explanation for this absence is that twitches in adults are somehow prevented from triggering spindle bursts in sensorimotor cortex. Given how reliably twitches activate the cerebral cortex in early development, this possibility requires that a mechanism emerges at some point between infancy and adulthood that gates twitch-triggered reafference. For example, there is evidence in adult cats of spinally mediated sensory gating that is stronger during REM sleep than during wakefulness [32]. However, even during REM sleep, this gating mechanism only partially blocks sensory feedback. Moreover, this study did not explicitly address twitch-related reafference. Therefore, it remains possible, if not likely, that twitches continue to trigger spindle bursts in the adult sensorimotor cortex.

## 2. Detecting Twitch-Related Spindle Bursts

**Figure 1:**
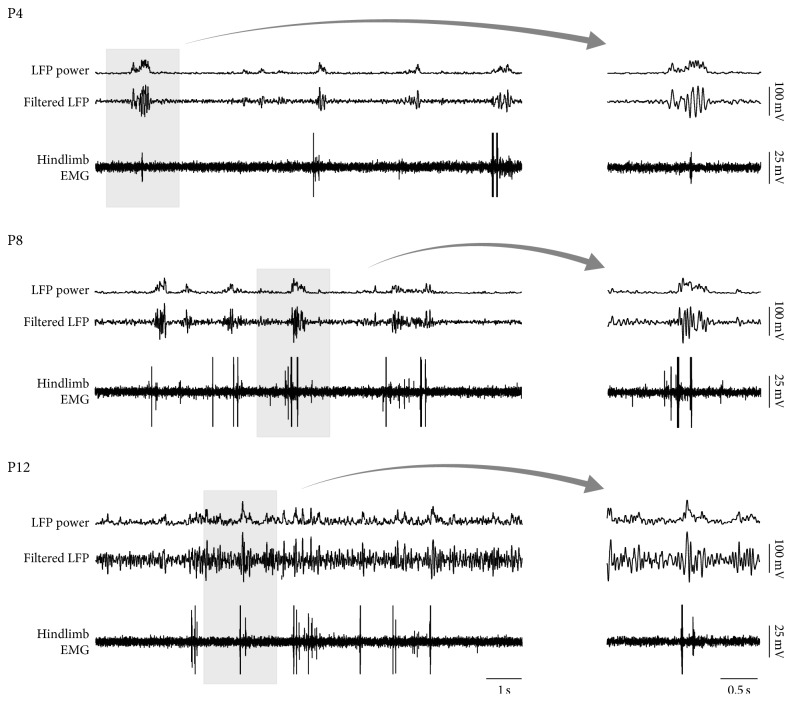
Spindle burst activity in motor cortex during REM sleep across the first two postnatal weeks in P4, P8, and P12 rats. At each age, the bottom trace is the hindlimb electromyogram (EMG), the middle trace is the band-pass filtered (1–40 Hz) local field potential (LFP) recorded from the hindlimb region of motor cortex, and the top trace is the root-mean-square (RMS; *τ* = 10 ms) of the LFP trace. The records in the gray boxes are expanded at right.

Figure 1 presents local field potentials (LFPs) in infants rats recorded from the hindlimb region of sensorimotor cortex in relation to twitches detected in the hindlimb electromyogram (EMG) (data from [11]). At postnatal days (P) 4 and 8–10, when background LFP activity is low, hindlimb twitches trigger easily discernible cortical spindle bursts. Indeed, because of the low background activity at these ages, visual inspection of the LFP alone—that is, without guidance provided by the hindlimb EMG—allows one to confidently state when a spindle burst occurred. Also, the LFP alone is sufficient to predict with high confidence when hindlimb twitches occurred.

In contrast, by P12, LFP background activity has increased such that spindle bursts are now much harder to detect. In this case, the LFP alone is not sufficient to predict with high confidence when hindlimb twitches occurred. Given this dramatic increase in background activity between P8–10 and P12, we expect spindle bursts to disappear further into the background over the ensuing days and weeks. But are spindle bursts actually disappearing?

**Figure 2:**
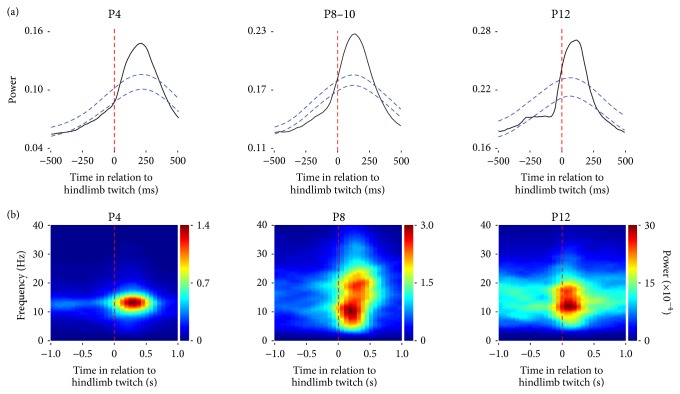
Spindle burst activity in relation to twitches in P4, P8–10, and P12 rats. (a) Waveform averages of local field potentials (LFPs) recorded from the hindlimb region of motor cortex (P4: *n* = 1,172 twitches across 6 pups; P8–10: *n* = 4,047 twitches across 11 pups; and P12: *n* = 789 twitches across 6 pups). Peak power in the LFP occurs after hindlimb twitches (dashed red lines). Acceptance bands (*p* < 0.05) are also shown (dashed blue lines). Adapted from Tiriac et al.,* Current Biology*, 2014 [11]. (b) Twitch-triggered time-frequency spectrograms of LFPs from individual P4 (168 twitches), P8 (294 twitches), and P12 (135 twitches) rats. Average peak power at spindle frequency (10–15 Hz) is prominent after hindlimb twitches (dashed red lines).

The precise relationship between twitches and spindle bursts is revealed using a twitch-triggered averaging method. In the case of sleeping infant rats at P4, P8–10, and P12, averaging LFP power in relation to hindlimb twitches reveals increased activity in the hindlimb region of sensorimotor cortex within ~150 ms of a twitch (Figure 2(a)). Time-frequency spectrograms reveal that these peaks in twitch-related power occur at spindle burst frequency (Figure 2(b)). Note that, at P4, the spectrogram displays a discrete “hotspot” immediately after hindlimb twitches. By contrast, at P12, there is increased background activity in the spindle frequency range; nonetheless, a concentrated twitch-related “hotspot” in spindle frequency is still apparent. These results suggest that, with increasing age, spindle bursts are harder to detect because they are obscured by increases in background LFP activity.

**Figure 3:**
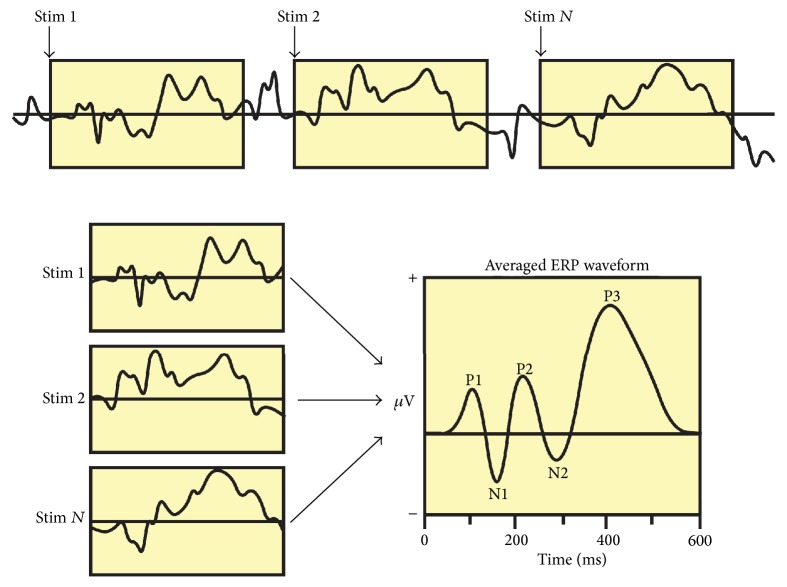
The event-related potential (ERP) technique. A raw EEG record is punctuated by the delivery of stimulus events (Stim  1, Stim  2,…, Stim  *N*) to the subject. The EEG data for each event-related window is then averaged to produce the ERP, which can exhibit several positive (e.g., P1) and negative (e.g., N1) components. Individual ERP components are thought to reflect various aspects of cortical processing. Using this method in adults during REM sleep—with individual twitches as the events while recording from a somatotopically related region of cortex—twitch-related spindle bursts within the activated EEG may be revealed. Figure used with permission of Steven J. Luck.

Event-triggered averaging, such as that illustrated in Figure 2, is routinely used to reveal hidden or obscured cortical events and oscillations. For example, cognitive neuroscientists use event-related potentials (ERPs) to reveal cortical activity associated with sensory, motor, or cognitive process (Figure 3; [33]). Because EEG electrodes reflect ongoing activity from thousands of neurons, the resulting signals are necessarily noisy. As a result, an individual event (e.g., a flash of light) may not be evident in the raw EEG signal. However, by averaging over many event-triggered trials, random noise that is not associated with neural processing of the stimulus cancels out, leaving behind a distinct and stereotyped cortical pattern—the ERP—that reflects underlying neural processes.

We propose that twitch-related spindle bursts have not yet been detected in the activated EEG of human adults during REM sleep because event-triggered averaging would be necessary to observe them. Undoubtedly, there are many published studies—in human and nonhuman adult animals—in which cortical activity recorded across behavioral states can be related to peripheral motor activity, including twitching (e.g., see [34]). Critically, to our knowledge, no study in adults has specifically used twitches for event-triggered averaging of cortical activity in somatotopically related areas of sensorimotor cortex.

## 3. Exploring the Functionality of Twitching and Associated Spindle Bursts across the Lifespan in Diverse Species

Species-typical and behaviorally relevant body parts—the bill of a platypus, the star appendage of a star-nosed mole, and the digits of a raccoon—have magnified representations in sensorimotor cortex [35]. The degree of magnification reflects the innervation of these peripheral morphological features as well as their use. If twitches are functionally important for the development and maintenance of sensorimotor circuits [8, 23], then we expect the quantity and patterning of twitching to reflect an appendage's innervation density, biomechanics, and behavioral importance across species [23, 36].

Like other primates, humans are a highly visual species that execute hundreds of thousands of saccadic eye movements (3 per second) during the day [37]. Interestingly, much like the skeletal muscles that control the limbs, the extraocular skeletal muscles that move the eyes twitch during REM sleep [38]. In fact, the resulting “rapid eye movements” give REM sleep its name. Moreover, much like the limbs of the body, proprioceptive reafference from eye movements in rats and humans is relayed to sensorimotor cortex [39, 40]. Since twitches of all skeletal muscles studied thus far trigger spindle bursts [8, 11, 21], we propose that rapid eye movements also trigger spindle bursts. And because rapid eye movements are a prominent feature of REM sleep in human adults, the sensorimotor system that controls eye movement could be an ideal model for exploring the contributions of spindle bursts to the calibration, maintenance, and repair of neural networks.

Whereas humans, which are diurnal, rely heavily on the visual system for spatial navigation, rats, which are nocturnal, rely heavily on the whiskers. The whiskers of rats and other rodents are controlled by an elaborate set of extrinsic and intrinsic striated muscles [41, 42]. Importantly, in infant rats, these muscles twitch during REM sleep and reafference from twitches activates the whisker thalamus and barrel cortex [21]. Spindle bursts are readily detected in the infant rat's barrel cortex [24], occurring immediately after twitching [43]; the developmental onset of whisker-related cortical activity could contribute to the refinement of the somatotopic map in barrel cortex [44]. Whisker twitching has also been observed in adult rats [45] but has not been systematically investigated. Therefore, similar to rapid eye movements in humans, we expect whisker twitching and associated spindle bursts to reflect the outsized role of this sensory modality in rats.

Extrapolating to other species, highly specialized sensorimotor appendages should exhibit relatively high rates of twitching. For example, the platypus uses a specialized bill to forage for food [46]. The bill contains a high density of electro- and mechanoreceptors and, consequently, a large proportion of the platypus' cerebral cortex is devoted to processing sensory input from the bill [46, 47]. As we would expect, platypuses exhibit vigorous twitches of the bill (as well as the eyes and limbs) during REM sleep, even as adults [48, 49].

The star-nosed mole uses a specialized set of 22 facial appendages that it uses like a “tactile fovea” to forage for food [50]. Similar to the bill of the platypus, the star appendages contain a high density of somatosensory receptors and, consequently, a large proportion of this animal's cerebral cortex is devoted to each appendage [50]. Although there is no information on the sleeping habits of the star-nosed mole, we predict that it will be found to exhibit high rates of twitching in the star appendages during REM sleep, as well as twitch-associated spindle bursts in sensorimotor cortex.

It has been argued that local changes in cortical slow-wave activity can be triggered by learning processes that differentially involve specific brain regions [51]. Similarly, after the development of a new waking skill or in response to changes in the sensory periphery (e.g., due to injury), perhaps twitches contribute to the process of learning, adaptation, or recovery of function. For example, in one study, human adults wore goggles during the day that decreased their visual field to 5 degrees [52]. When these subjects were observed over several days during sleep, the duration of REM sleep was unaffected, but the frequency and amplitude of rapid eye movements increased significantly. Because the changes in rapid eye movements were a transient response to a waking perturbation of visual experience, these findings suggest to us that twitching—in this case in the form of rapid eye movements—contributes to the process of visual adaptation. This experimental approach—that is, the manipulation of waking motor experience in human and non-human subjects—could provide the opportunity to explore the contributions of twitching and twitch-related spindle bursts to learning and adaptation in health and disease.

## 4. Conclusions

Until recently, twitches were widely perceived as functionless byproducts of dreaming [23], providing little reason to search for their neural consequences. Only with the discovery of twitch-related neural activity in infant rats, it has become apparent that twitches could play a functional role in sensorimotor development, akin to spontaneous activity in other sensory systems. It is the persistence of twitching into adulthood, in humans and other mammals, that raises the intriguing possibility that twitching has much more to reveal to us about its functional contributions to neural plasticity within the sensorimotor system.

Although the idea that twitch-related spindle bursts persist into adulthood is speculative, it can easily be tested using new—or even perhaps existing—recordings. If evidence of twitch-related spindle bursts is found, there will be a strong basis for expanding our understanding of the functions of spindle activity during non-REM sleep—about which we currently know a lot—to include REM sleep as well.
